# Altered Brain Function Activity in Patients With Dysphagia After Cerebral Infarction: A Resting-State Functional Magnetic Resonance Imaging Study

**DOI:** 10.3389/fneur.2022.782732

**Published:** 2022-07-14

**Authors:** Lei Li, Jiayu Liu, Fenxiong Liang, Haidong Chen, Rungen Zhan, Shengli Zhao, Tiao Li, Yongjun Peng

**Affiliations:** ^1^Department of Nuclear Medicine, Zhuhai People's Hospital (Zhuhai Hospital Affiliated With Jinan University), Zhuhai, China; ^2^Department of Neurosurgery, Peking University People's Hospital, Beijing, China; ^3^Department of Radiology, Zhuhai People's Hospital (Zhuhai Hospital Affiliated With Jinan University), Zhuhai, China; ^4^Department of Rehabilitation Medicine, Zhuhai People's Hospital (Zhuhai Hospital Affiliated With Jinan University), Zhuhai, China

**Keywords:** dysphagia, cerebral infarction, rs-fMRI, ReHo, fALFF

## Abstract

**Objective:**

Dysphagia after cerebral infarction (DYS) has been detected in several brain regions through resting-state functional magnetic resonance imaging (rs-fMRI). In this study, we used two rs-fMRI measures to investigate the changes in brain function activity in DYS and their correlations with dysphagia severity.

**Method:**

In this study, a total of 22 patients with DYS were compared with 30 patients without dysphagia (non-DYS) and matched for baseline characteristics. Then, rs-fMRI scans were performed in both groups, and regional homogeneity (ReHo) and fractional amplitude of low-frequency fluctuation (fALFF) values were calculated in both groups. The two-sample *t*-test was used to compare ReHo and fALFF between the groups. Pearson's correlation analysis was used to determine the correlations between the ReHo and fALFF of the abnormal brain regions and the scores of the Functional Oral Intake Scale (FOIS), the Standardized Bedside Swallowing Assessment (SSA), the Videofluoroscopic Swallowing Study (VFSS), and the Penetration-Aspiration Scale (PAS).

**Results:**

Compared with the non-DYS group, the DYS group showed decreased ReHo values in the left thalamus, the left parietal lobe, and the right temporal lobe and significantly decreased fALFF values in the right middle temporal gyrus and the inferior parietal lobule. In the DYS group, the ReHo of the right temporal lobe was positively correlated with the SSA score and the PAS score (*r* = 0.704, *p* < 0.001 and *r* = 0.707, *p* < 0.001, respectively) but negatively correlated with the VFSS score (*r* = −0.741, *p* < 0.001). The ReHo of the left parietal lobe was positively correlated with SSA and PAS (*r* = 0.621, *p* = 0.002 and *r* = 0.682, *p* < 0.001, respectively) but negatively correlated with VFSS (*r* = −0.679, *p* = 0.001).

**Conclusion:**

The changes in the brain function activity of these regions are related to dysphagia severity. The DYS group with high ReHo values in the right temporal and left parietal lobes had severe dysphagia.

## Introduction

Stroke is one of the leading causes of dysphagia, and dysphagia is an independent risk factor for prognosis in patients following stroke (1). Approximately 37–78% of poststroke patients have dysphagia (2). Dysphagia can occur not only in patients with brain stem stroke but also in patients with cerebral hemisphere stroke (3). Patients with dysphagia are at risk of dehydration and malnutrition because of the difficulty in ingesting food due to disruption of the swallowing process (4). Some patients with dysphagia develop respiratory tract infections and aspiration pneumonia, and the infection-related death rate in such patients reaches 50% (5). Dysphagia is not solely a physical hazard; it can have mental and social effects, leading to depression and a lower quality of life (6). However, the neural mechanism of dysphagia is still unclear. Dysphagia was at one time thought to be an automatic response, one that occurs primarily at the brainstem level (7). Recently, some studies have reported that dysfunction of the swallowing motor areas or their connection to the brainstem might be the cause of swallowing dysfunction (8).

Resting-state functional magnetic resonance imaging (rs-fMRI) is a rapidly developing and non-invasive neurofunctional imaging technique with high temporal and spatial resolution. It is an important method for the study of brain function. Multiple functional neuroimaging studies have shown that, in addition to the brainstem, the cerebral cortex (including the insula and the postcentral gyrus) and the subcortical structures (including the basal ganglia) are associated with dysphagia in stroke patients (9–11). The cerebral cortex also plays an important role in the regulation of swallowing (9). While many studies have shown deficits in brain function in patients with dysphagia following stroke, the results have been inconsistent, especially regarding the direction of activation.

The fractional amplitude of low-frequency fluctuation (fALFF) and regional homogeneity (ReHo) are the major rs-fMRI metrics used for studying local brain function activity (12). fALFF helps identify specific local brain regions with abnormal oxygen level-dependent signals and activity, and ReHo can reflect the consistency of brain activity in a time series (13). To date, fALFF and ReHo have not been investigated for their associations with the severity of dysphagia after cerebral infarction (DYS). In this study, we used these rs-fMRI measures to investigate the changes in brain function activity in DYS and analyzed their correlations with dysphagia severity.

## Methods

### Participants

This case-control study was conducted from January 2019 to November 2020. Twenty-two patients with DYS were recruited from the Department of Rehabilitation Medicine of Zhuhai People's Hospital. The inclusion criteria were as follows: (1) One-sided cerebral infarction had been diagnosed by two radiologists based on nerve injury and computed tomography (CT) or MRI of the head (with a duration of <3 months since cerebral infarction). (2) The patient had a Functional Oral Intake Scale (FOIS) score ≤ 4 (14), as assessed by the associate chief physician of the rehabilitation department (15). (3) Baseline mental status at the time of rs-fMRI would not induce or worsen dysphagia. The patients were instructed to avoid alcohol and caffeinated beverages in the 24 h before rs-fMRI. The exclusion criteria were as follows: (1) lack of capacity to consent due to neuropsychological, linguistic, or psychiatric disorders; (2) head motion correction involving translation more than 3 mm or rotation more than 3°; (3) brainstem infarction (as this study was focused on the role of cortical and subcortical structures in swallowing control); (4) prior cerebrovascular disease; (5) use of an electrically sensitive biomedical device (cardiac pacemaker or cochlear implant); (6) metal clips in the brain, (7) pneumonia at the time of enrolment; (8) alcohol and caffeinated beverages were used on the day of the scan. Thirty patients without dysphagia (non-DYS) that matched to the DYS group in terms of baseline characteristics were selected from the Department of Radiology as controls.

All subjects were right-handed and volunteered to participate in the study. This study was approved by the Research Ethics Committee of Zhuhai People's Hospital of Guangdong Province, China. All subjects provided signed informed consent.

### MRI Data Acquisition

MRI scans were performed using a GE Signa HDxt 3.0T MR scanner (GE Healthcare, Chicago, IL, USA) and an eight-channel receiver array head coil. Fillers and earplugs were used to reduce head movement and scanner noise. The subjects were instructed to close their eyes, rest, avoid thinking about anything, and avoid head movement during the scan. First, high-resolution 3D T1-weighted structural images were acquired with the following parameters: repetition time (TR) = 600 ms, echo time (TE) = 17 ms, field of view (FOV) = 200 × 200 mm, slice thickness = 0.6 mm, gap = 0 mm, and matrix= 256 × 256. Diffusion-weighted imaging (DWI) was performed using the following imaging parameters: axial image, echoplanar imaging sequence, *b*-values = 0 and 1,000 s/mm^2^, TR = 2,501 ms, TE = 98 ms, flip angle = 90°, FOV = 230 × 230 mm, matrix = 152 × 122, and slice thickness = 6 mm. The axial rs-fMRI image was obtained using a gradient echo-planar imaging sequence with the following parameters: TR = 2,000 ms, TE = 30 ms, slices = 23, slice thickness = 4 mm, gap = 0 mm, flip angle = 90°, FOV = 240 mm × 240 mm, and matrix = 64 × 64. The scanning time is within 5–7 min. After each MRI scan, the subject was asked whether he or she had been asleep or distracted during the scan for the exclusion of unqualified subjects.

### Data Collection

**Figure 1:**
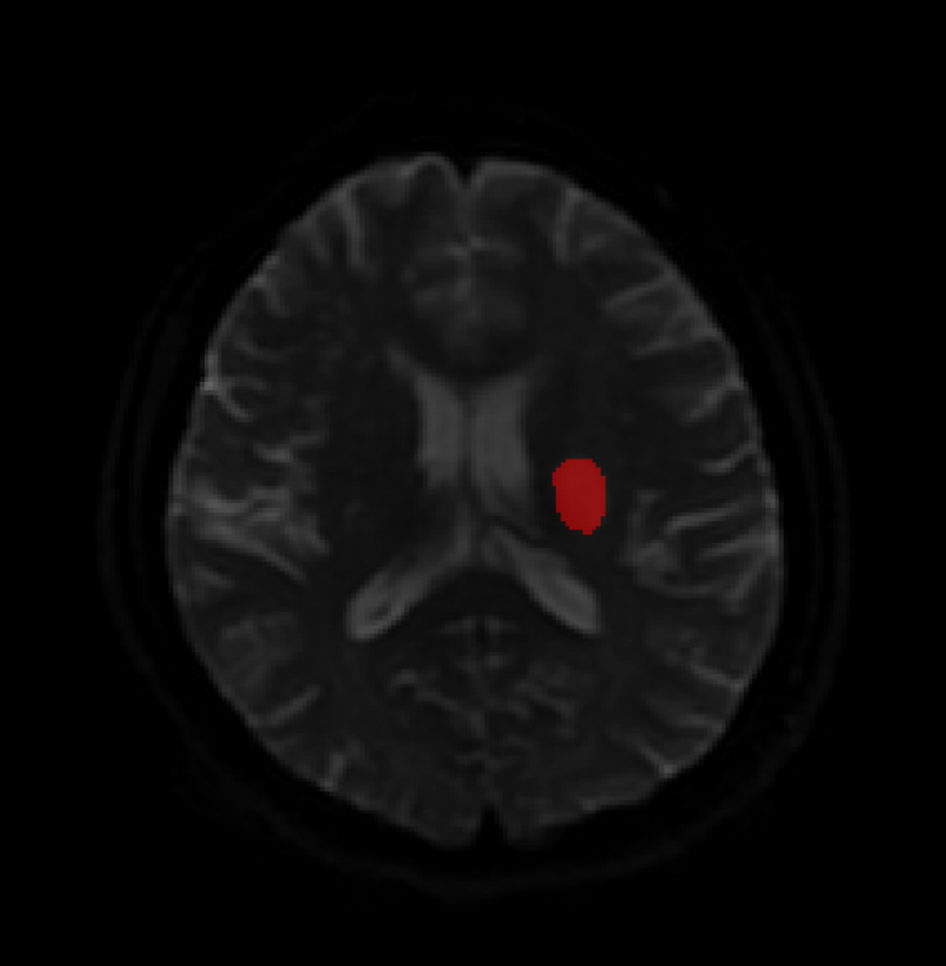
An example of infarct volume views on diffusion-weighted imaging (DWI).

Baseline data and medical histories were obtained from patient medical records. The baseline data included sex, age, diabetes status, hypertension status, duration since stroke onset, the National Institute of Health Stroke Scale (NIHHS) score (16), affected hemisphere, lesion location, and infarct volume. DWI contributed significantly to estimating infarct volume. Infarct lesions were manually segmented on admission DWI *via* ITK-Snap software, version 3.0 (www.itk-snap.org). An intensity filter was used to augment the selection and segmentation of the DWI-hyperintense lesions. The lesion volumes were also calculated using the aforementioned software (Figure 1) (17).

Dysphagia was evaluated using FOIS and the Standardized Bedside Swallowing Assessment (SSA), Videofluoroscopic Swallowing Study (VFSS), and Penetration-Aspiration Scale (PAS), for those who were experiencing swallowing dysfunction after cerebral infarction. Different types of dysphagia were assessed using the VFSS (18–20). The severity of dysphagia was assessed using the PAS, which is an 8-point scale that evaluates airway invasion based on the VFSS, by a rehabilitation doctor after cerebral infarction (21).

### Functional Data Preprocessing

Data preprocessing was performed using the Data Processing Assistant for the rs-fMRI (DPARSF) tool kit (http://rfmri.org/DPARSF) in the MATLAB R2016a programming environment (The MathWorks, Natick, MA) (22). The specific processing steps entailed the following: (1) conversion of the DICOM images to the NIFIT format; (2) filtering out of the first 10 time points; (3) time correction; (4) head motion correction and the exclusion of translation values more than 3 mm or rotation values more than 3°; (5) space standardization, brain normalization to Echo-Planar Imaging (EPI) template in the Montreal Neurological Institute (MNI) space; (6) removal of linear drift and nuisance covariates; and (7) spatial smoothing. Spatial smoothing was performed to reduce spatial noise and local anatomic structure artifacts, and the value of full width at half maximum (FWHM) was 6 mm.

### ReHo and fALFF Analyses

Regional homogeneity was calculated as Kendall's concordance coefficient (KCC), which reflects the temporal consistency of neural activity in a region of the brain (13). ReHo maps were normalized by dividing the KCC among each voxel by the global mean ReHo value. The resulting data were spatially smoothed by convolution with a 4 mm FWHM Gaussian kernel.

An index named amplitude of low-frequency fluctuation (ALFF) of the rs-fMRI signal has been suggested to reflect the intensity of regional spontaneous brain activity. In this study, the power spectrum was acquired using fast Fourier transformation to convert each voxel's time series into the frequency domain. Then, each frequency of the power spectrum was square-root-transformed at each voxel. The averaged square root of the frequency range of 0.01–0.1 Hz was defined as the ALFF value. fALFF analysis was performed by smoothing *via* a Gaussian function with 4 mm FWHM. The value of ALFF in this range was added to obtain the total ALFF value, and the fALFF value was obtained by dividing the total value by the full-band amplitude from 0.01 to 0.25 Hz (23).

### Statistical Analysis

SPSS 25.0 statistical software (IBM Corp, Armonk, NY, USA) was used for the data analysis. Numerical variables are expressed as the mean ± standard deviation. Qualitative variables are described by absolute values of cases in different groups. The statistical significance of differences between the quantitative variables was assessed by the χ^2^ test with Yates' correction or by Fisher's exact test as appropriate. Student's *t*-tests were performed to evaluate data that followed a normal distribution. A *p* < 0.05 was considered statistically significant.

Two-sampled *t*-tests were performed to analyze the differences between the two groups in ReHo and fALFF values. Age, sex, and the head motion parameters of each subject were included as covariates. The resulting statistical map was corrected through multiple comparison corrections to a significance level of *p* < 0.05. The multiple comparison correction was performed using Gaussian random field (GRF) theory correction with an individual voxel threshold of *p* < 0.001 and cluster-level *p* < 0.05. The results were displayed *via* the ch2.nii template of MRIcron software, which is the standard and well-known Colin27 template. The ReHo and fALFF values were extracted from the above-differentiated brain regions, and correlation analysis was performed for the FOIS, SSA, VFSS, and PAS scores. The relationship between the values of fALFF and ReHo was studied *via* Pearson's correlation analysis. A *p* < 0.05 was considered statistically significant. Multiple comparison correction was performed using the false discovery rate criterion of an individual voxel threshold, *p* < 0.001, and at cluster-level, *p* < 0.05.

## Results

### Clinical Features and Dysphagia Examination

Table 1 shows the demographic data of the subjects. No significant differences were observed in sex, age, diabetes status, hypertension status, duration since stroke onset, the NIHHS score, affected hemisphere, lesion location, or infarct volume between the two groups (*p* > 0.05).

**Table 1:** Clinical characteristics of the groups.

**Characteristic**	**Dys group (*n* = 22)**	**Non-DYS group (*n* = 30)**	***P*-value**
Age (mean ± SD, year)	60.0 ± 11.38	54.57 ± 10.5	0.086
Sex (female, %)	10 (45.46)	13 (35.00)	0.782
Diabetes mellitus (*n*, %)	7 (31.81)	10 (33.33)	0.267
Hypertension (*n*, %)	13 (59.10)	17 (56.67)	0.166
Duration since stroke onset-days	17.18 ± 10.03	18.04 ± 9.79	0.663
NIHSS (score)	9.22 ± 3.44	8.93 ± 3.43	0.762
Aff.Hem (L, %)	16 (72.72)	15 (50.00)	0.405
**Lesion location (** ***n*** **, %)**			
Cerebral lobe	12 (54.54)	15 (50.00)	0.355
Basal ganglia	6 (27.28)	13 (43.33)	
Thalamus	4 (18.18)	2 (6.67)	
Infarct lesion volumes (mean ± SD, ml)	13.42 ± 2.01	14.97 ± 3.98	0.101
FOIS (mean ± SD, score)	2.36 ± 0.50	/	
SSA (mean ± SD, score)	33.18 ± 1.94	/	
VFSS (mean ± SD, score)	15.18 ± 1.78	/	
PAS (mean ± SD, score)	17.82 ± 3.70	/	

### ReHo and fALFF Analyses

**Figure 2:**
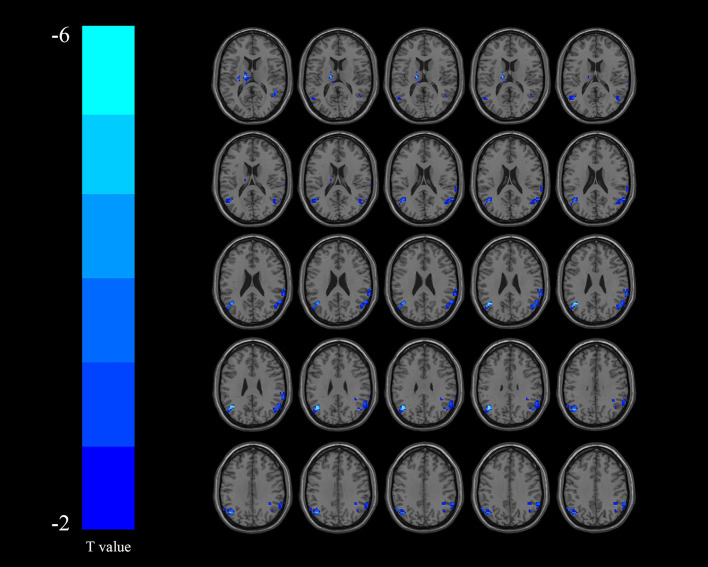
Regions exhibiting differences in regional homogeneity (ReHo) between the dysphagia after cerebral infarction (DYS) and non-DYS groups [*p* < 0.001 corrected by Gaussian random field (GRF)]. Cooler colors indicate significantly lower ReHo values in the DYS group.

**Figure 3:**
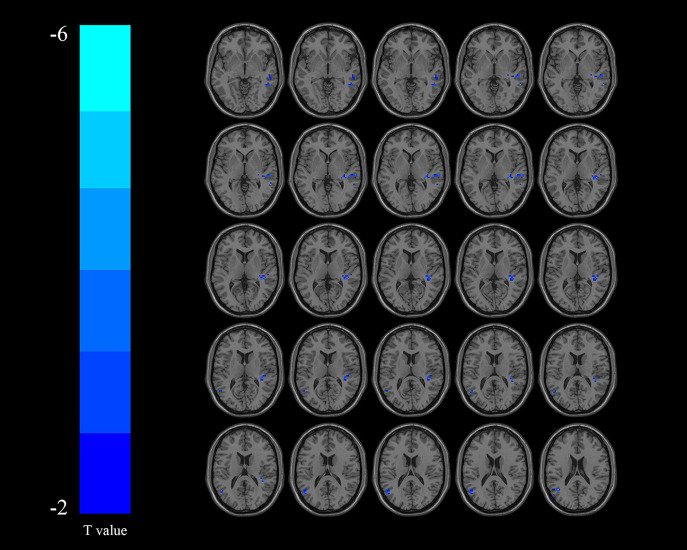
Regions exhibiting differences in fractional amplitude of low-frequency fluctuation (fALFF) between the dysphagia after cerebral infarction (DYS) and non-DYS groups [*p* < 0.001 corrected by Gaussian random field (GRF)]. Cooler colors indicate significantly lower regional homogeneity (ReHo) values in the DYS group.

Compared with the non-DYS group, the DYS group showed decreased ReHo values in the left thalamus, the left parietal lobe, and the right temporal lobe (Table 2 and Figure 2) and significantly decreased fALFF values in the right middle temporal gyrus and the inferior parietal lobe (Table 3 and Figure 3; corrected voxel-level, *p* < 0.001 and cluster-level, *p* < 0.05).

**Table 2:** Regions showing significant differences in ReHo values between DYS and non-DYS.

**AAL cluster lables**	**Cluster size**	**Peak MNI coordinate**			**Peak-Tscore**
		**X**	**Y**	**Z**	
Left thalamus	174	−15	−15	12	−5.12
Left parietal Lobe	271	57	−57	24	−3.59
Right temporal Lobe	184	−45	−63	30	−5.58

**Table 3:** Regions showing significant differences in fALFF values between DYS and non-DYS.

**AAL cluster lables**	**Cluster size**	**Peak MNI coordinate**			**Peak-Tscore**
		**X**	**Y**	**Z**	
Right middle temporal gyrus	85	57	−45	0	−3.65
Inferior parietal lobule	84	−51	−60	39	−4.8

### Correlation Analysis

**Figure 4:**
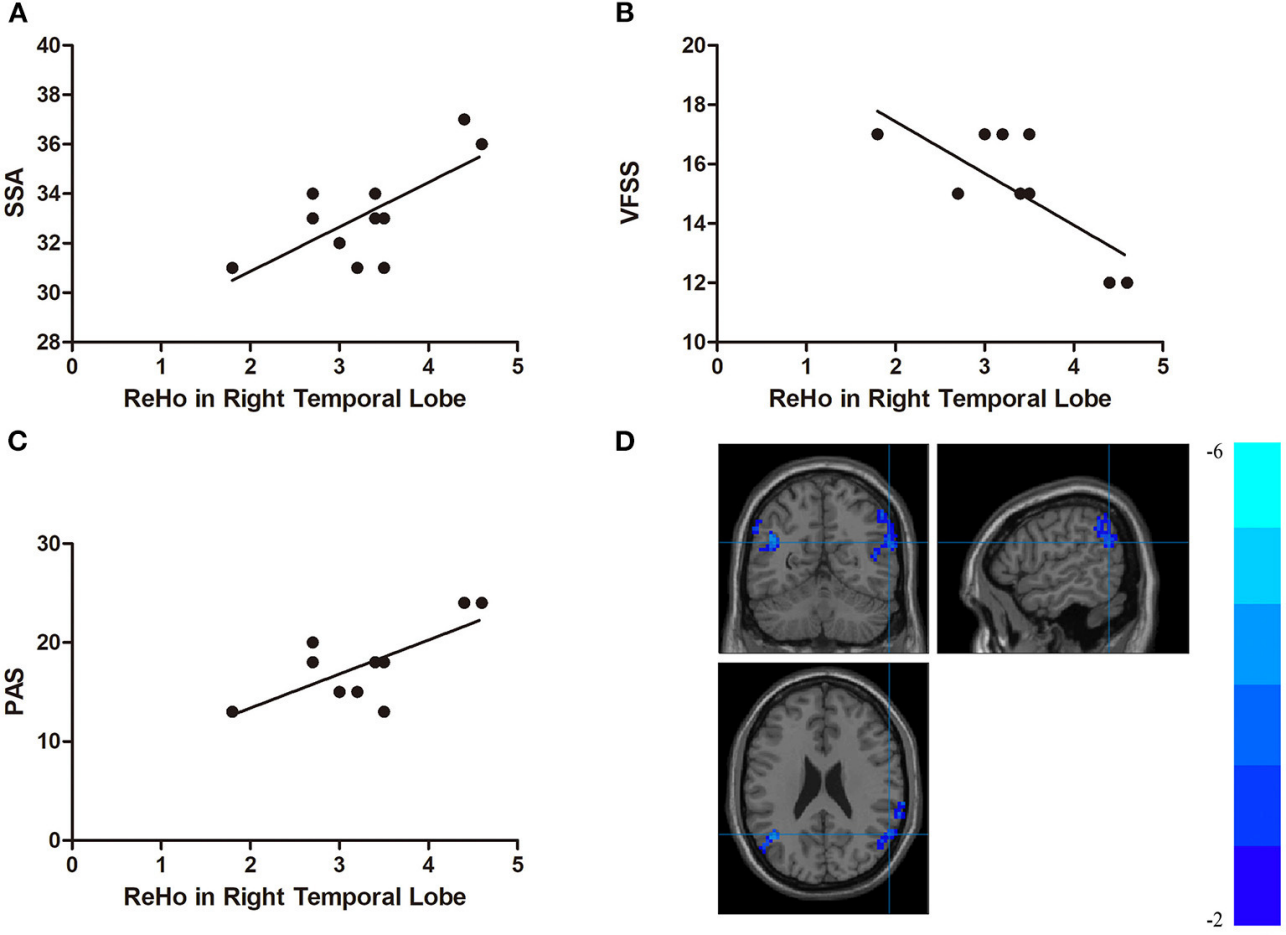
**(A)** Correlations between the Standardized Bedside Swallowing Assessment (SSA) score and regional homogeneity (ReHo) in the right temporal lobe; **(B)** Correlations between the Videofluoroscopic Swallowing Study (VFSS) score and decreased ReHo in the right temporal lobe; **(C)** Correlations between the Penetration-Aspiration Scale (PAS) and decreased ReHo in the right temporal lobe; **(D)** Regions exhibited in the right temporal lobe [*p* < 0.001 corrected by the Gaussian random field (GRF)].

**Figure 5:**
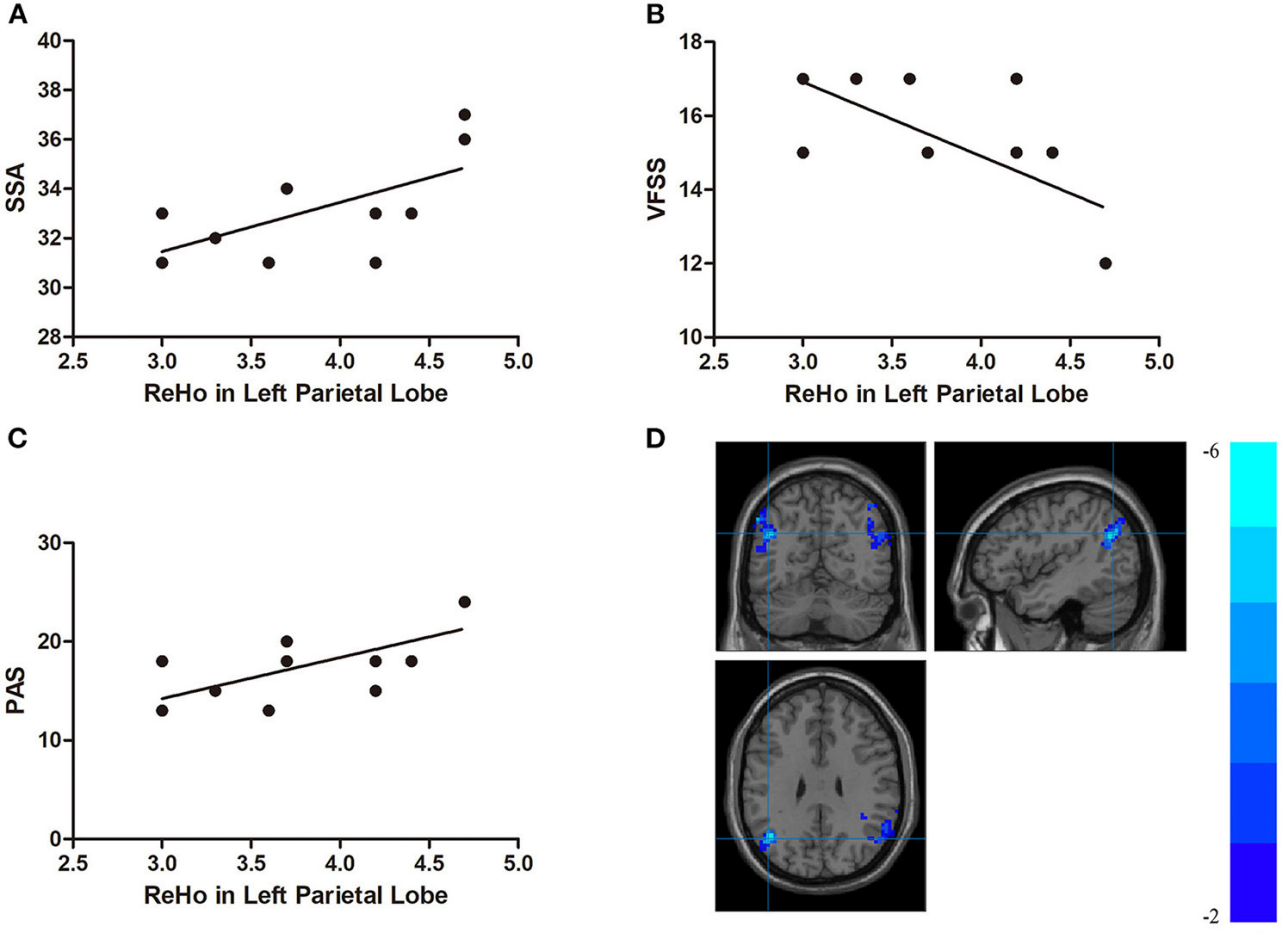
**(A)** Correlations between the Standardized Bedside Swallowing Assessment (SSA) score and decreased ReHo in the left parietal lobe; **(B)** Correlations between Videofluoroscopic Swallowing Study (VFSS) score and decreased ReHo in the left parietal lobe; **(C)** Correlations between Penetration-Aspiration Scale (PAS) score and decreased ReHo in the left parietal lobe; **(D)** Regions exhibited in the left parietal lobe [*p* < 0.001 corrected by Gaussian random field (GRF)].

In the DYS group, the ReHo of the right temporal lobe was positively correlated with the SSA score and the PAS score (*r* = 0.704, *p* < 0.001 and *r* = 0.707, *p* < 0.001, respectively) but negatively correlated with VFSS (*r* = −0.741, *p* < 0.001; Figure 4). The ReHo of the left parietal lobe was positively correlated with SSA and PAS (*r* = 0.621, *p* = 0.002 and *r* = 0.682, *p* < 0.001, respectively) but negatively correlated with VFSS (*r* = −0.679, *p* = 0.001; Figure 5). No significant correlation was found between the fALFF of the right middle temporal gyrus or the inferior parietal lobule and any of the scores.

## Discussion

Swallowing activity is a complex sensory-motor process simultaneously involving visual, auditory, olfactory, and gustatory sensors and different regions, such as the cerebral cortex and the subcortex (24). Dysphagia often occurs in patients with stroke, and changes in brain function are the pathological basis of dysphagia. In this study, we recruited patients with DYS and patients without DYS matched for baseline characteristics, and we used fALFF and ReHo to explore the changes in local brain functional activity and the correlations of these metrics with the degree of dysphagia in patients with DYS to further understand the neural mechanism of DYS. The results showed that relative to the non-DYS patients, the patients with DYS had significantly decreased brain function activity in the left thalamus, the left parietal lobe, the right temporal lobe, the right middle temporal gyrus, and the inferior parietal lobule. In addition, the ReHo values of the right temporal and parietal lobes were negatively correlated with the degree of dysphagia in the DYS group. These region-specific changes in brain function activity may play a key role in DYS.

ReHo, a new metric for analyzing brain signals by rs-fMRI, was first proposed by Zang et al. (13). The theoretical basis is that, under certain conditions, there is a strong temporal consistency between a brain voxel and the surrounding voxels. A decreasing ReHo value indicates a decreasing consistency of neuronal activities. Furthermore, an abnormal ReHo value indicates that the activity of neurons in the brain region is abnormal, which in turn indicates the disorder of the interaction between neurons or pathological changes of the neurons themselves. Therefore, ReHo analysis is a method that targets the functional activity of the local brain. Its advantage lies in its ability to locate different brain regions, and it has been widely used in the study of various neuropsychiatric diseases (25). In this study, abnormalities in local brain functional activity in several brain regions, mainly in the left thalamus, the left parietal lobe, and the right temporal lobe, in the DYS group compared with the non-DYS group were detected through ReHo analysis. When stroke patients with dysphagia attempt to perform swallowing activities, most of the cerebral cortical activation related to swallowing function is decreased, so they cannot complete swallowing activities (26). The swallowing preparation period and the oral period require attention. Patients with attention disorders are easily affected by interference in eating (27). The parietal lobe participates in the composition of the attention network (28), which is related to the spatial positioning of sensory information. The parietal lobe and other brain regions play important roles in the regulation of neural activities triggered by attention to sensory information (29). In addition, the primary sensorimotor cortex is the most consistently and strongly activated brain area during autonomic swallowing. Soros et al. (30) proposed that the primary sensorimotor cortex is involved in the execution of autonomic movements and may be related to the initiation of swallowing. Previous studies have demonstrated sensory integration in the parietal temporal and parietal lobes (31, 32), and the temporal lobe is also involved in the processing of sensory information (33). In a study of eight adults without dysphagia, Kern et al. (34) found that reflex swallowing is regulated by the primary sensorimotor cortex. The thalamus acts as a relay station connecting different subcortical regions to the cerebral cortex, through which all sensory pathways except smell are projected back to the cortical region. The results of our study showed that temporal and thalamic changes in brain function activity may play a key role in DYS. In the current study, the ReHo values of the right temporal lobe and the left parietal lobe were negatively correlated with the degree of dysphagia. According to our findings, the local neural activity abnormalities in these regions are closely related to the severity of maternal dysphagia. The fALFF reflects the intensity of spontaneous activity in brain regions. In this study, the fALFF values of patients with DYS were significantly decreased in the right middle temporal gyrus and the inferior parietal lobe, which also demonstrates the importance of these brain regions in DYS. However, in this study, brain function activity (ReHo or fALFF) in brain regions was only unilateral. The asymmetry of the distribution of swallowing centers in both cerebral hemispheres may be the cause of dysphagia resulting from unilateral stroke. When the dominant swallowing center is damaged, the non-dominant swallowing center on the healthy side is insufficient to maintain normal swallowing (35). Normal swallowing requires the bilateral deglutition pathway. After the unilateral stroke, the healthy side cannot control the deglutition activity alone. Regarding the anatomical and physiological basis of this activity, the peripheral sensory that afferent fibers usually project to the bilateral primary sensory cortex emit connective fibers to the primary motor cortex and finally emanate from the bilateral cortical medulla tracts (36).

However, brainstem infarction is one of the most common causes of dysphagia after infarction. This study did not include patients with brainstem infarction, as this study was focused on the functional changes in cortical and subcortical areas. In the future, we will study patients with the cerebral cortex and brainstem infarction and explore the differences in functional connectivity. In addition, due to the limited number of patients, the type and severity of dysphagia were not taken into account, and we will concentrate on this in our upcoming study. At last, our findings are early results (mean: 17 + 10 days) and we will further study the long-term results and post-treatment changes in the future.

## Conclusion

In this study, we used ReHo and fALFF of rs-fMRI to investigate the changes in brain function activity in DYS and their correlations with dysphagia severity. Changes in the brain function activity of the thalamus, the temporal lobe, and the parietal lobe are related to dysphagia severity. The DYS group with high ReHo values in the right temporal and left parietal lobes had severe dysphagia.

## Data Availability Statement

The raw data supporting the conclusions of this article will be made available by the authors, without undue reservation.

## Ethics Statement

The studies involving human participants were reviewed and approved by the Research Ethics Committee of Zhuhai People's Hospital of Guangdong Province, China. The patients/participants provided their written informed consent to participate in this study.

## Author Contributions

LL and JL: literature search, study design, data collection, data interpretation, and writing. FL: literature search, data analysis, data interpretation, and writing. YP: study design and feedback on the manuscript. TL: data collection and feedback on the manuscript. HC, RZ, and SZ: MRI scan. All authors contributed to the article and approved the submitted version.

## Conflict of Interest

The authors declare that the research was conducted in the absence of any commercial or financial relationships that could be construed as a potential conflict of interest.

## Publisher's Note

All claims expressed in this article are solely those of the authors and do not necessarily represent those of their affiliated organizations, or those of the publisher, the editors and the reviewers. Any product that may be evaluated in this article, or claim that may be made by its manufacturer, is not guaranteed or endorsed by the publisher.

## References

[1] Smithard DG O'Neill PA Parks C Morris J. Complications and outcome after acute stroke. Does dysphagia matter? Stroke. (1996) 27:1200–4. 10.1161/01.STR.27.7.1200 8685928

[2] Martino R Foley N Bhogal S Diamant N Speechley M Teasell R. Dysphagia after stroke: incidence, diagnosis, and pulmonary complications. Stroke. (2005) 36:2756–63. 10.1161/01.STR.0000190056.76543.eb 16269630

[3] Kruger E Teasell R Salter K Foley N Hellings C. The rehabilitation of patients recovering from brainstem strokes: case studies and clinical considerations. Top Stroke Rehabil. (2007) 14:56–64. 10.1310/tsr1405-56 17901016

[4] Sarabia-Cobo CM Perez V de Lorena P Dominguez E Hermosilla C Nunez MJ. The incidence and prognostic implications of dysphagia in elderly patients institutionalized: a multicenter study in Spain. Appl Nurs Res. (2016) 30:e6–9. 10.1016/j.apnr.2015.07.001 26235494

[5] Cabre M Serra-Prat M Palomera E Almirall J Pallares R Clave P. Prevalence and prognostic implications of dysphagia in elderly patients with pneumonia. Age Ageing. (2010) 39:39–45. 10.1093/ageing/afp100 19561160

[6] Puisieux F D'Andrea C Baconnier P Bui-Dinh D Castaings-Pelet S Crestani B. Swallowing disorders, pneumonia and respiratory tract infectious disease in the elderly. Rev Mal Respir. (2011) 28:e76–93. 10.1016/j.rmr.2011.09.007 22099417

[7] Osawa A Maeshima S Matsuda H Tanahashi N. Functional lesions in dysphagia due to acute stroke: discordance between abnormal findings of bedside swallowing assessment and aspiration on videofluorography. Neuroradiology. (2013) 55:413–21. 10.1007/s00234-012-1117-6 23160534

[8] Hamdy S Aziz Q Thompson DG Rothwell JC. Physiology and pathophysiology of the swallowing area of human motor cortex. Neural Plast. (2001) 8:91–7. 10.1155/NP.2001.91 11530891 PMC2565392

[9] Horner J Buoyer FG Alberts MJ Helms MJ. Dysphagia following brain-stem stroke. Clinical correlates and outcome. Arch Neurol. (1991) 48:1170–3. 10.1001/archneur.1991.00530230078026 1953404

[10] Daniels SK Foundas AL. The role of the insular cortex in dysphagia. Dysphagia. (1997) 12:146–56. 10.1007/PL00009529 9190100

[11] Cola MG Daniels SK Corey DM Lemen LC Romero M Foundas AL. Relevance of subcortical stroke in dysphagia. Stroke. (2010) 41:482–6. 10.1161/STROKEAHA.109.566133 20093638

[12] Che K Mao N Li Y Liu M Ma H Bai W. Altered spontaneous neural activity in peripartum depression: a resting-state functional magnetic resonance imaging study. Front Psychol. (2020) 11:656. 10.3389/fpsyg.2020.00656 32346374 PMC7172032

[13] Zang Y Jiang T Lu Y He Y Tian L. Regional homogeneity approach to fMRI data analysis. Neuroimage. (2004) 22:394–400. 10.1016/j.neuroimage.2003.12.030 15110032

[14] Crary MA Mann GD Groher ME. Initial psychometric assessment of a functional oral intake scale for dysphagia in stroke patients. Arch Phys Med Rehabil. (2005) 86:1516–20. 10.1016/j.apmr.2004.11.049 16084801

[15] Battel I Calvo I Walshe M. Cross-cultural validation of the italian version of the functional oral intake scale. Folia Phoniatr Logop. (2018) 70:117–23. 10.1159/000490792 30089299

[16] Kwah LK Diong J. National Institutes of Health Stroke Scale (NIHSS). J Physiother. (2014) 60:61. 10.1016/j.jphys.2013.12.012 24856948

[17] Arca G Arnaez J Agut T Nunez C Stephan-Otto C Valls A. Neuron-specific enolase is correlated with lesion topology, relative infarct volume and outcome of symptomatic NAIS. Arch Dis Child Fetal Neonatal Ed. (2020) 105:132–7. 10.1136/archdischild-2018-316680 31201253

[18] Rosenbek JC Robbins JA Roecker EB Coyle JL Wood JL. A penetration-aspiration scale. Dysphagia. (1996) 11:93–8. 10.1007/BF00417897 8721066

[19] Hiorns MP Ryan MM. Current practice in paediatric videofluoroscopy. Pediatr Radiol. (2006) 36:911–9. 10.1007/s00247-006-0124-3 16552584

[20] Park YH Bang HL Han HR Chang HK. Dysphagia screening measures for use in nursing homes: a systematic review. J Korean Acad Nurs. (2015) 45:1–13. 10.4040/jkan.2015.45.1.1 25743729

[21] Luan S Wu SL Xiao LJ Yang HY Liao MX Wang SL. Comparison studies of ultrasound-guided botulinum toxin injection and balloon catheter dilatation in the treatment of neurogenic cricopharyngeal muscle dysfunction. NeuroRehabilitation. (2021) 49:629–39. 10.3233/NRE-210113 34806624

[22] Chao-Gan Y Yu-Feng Z. DPARSF: a MATLAB toolbox for “pipeline” data analysis of resting-state fMRI. Front Syst Neurosci. (2010) 4:13. 10.3389/fnsys.2010.00013 20577591 PMC2889691

[23] Zou QH Zhu CZ Yang Y Zuo XN Long XY Cao QJ. An improved approach to detection of amplitude of low-frequency fluctuation (ALFF) for resting-state fMRI: fractional ALFF. J Neurosci Methods. (2008) 172:137–41. 10.1016/j.jneumeth.2008.04.012 18501969 PMC3902859

[24] Toogood JA Smith RC Stevens TK Gati JS Menon RS Theurer J. Swallowing preparation and execution: insights from a delayed-response functional magnetic resonance imaging (fMRI) study. Dysphagia. (2017) 32:526–41. 10.1007/s00455-017-9794-2 28361202

[25] Wang T Li S Jiang G Lin C Li M Ma X. Regional homogeneity changes in patients with primary insomnia. Eur Radiol. (2016) 26:1292–300. 10.1007/s00330-015-3960-4 26350539

[26] Mihai PG Otto M Domin M Platz T Hamdy S Lotze M. Brain imaging correlates of recovered swallowing after dysphagic stroke: a fMRI and DWI study. Neuroimage Clin. (2016) 12:1013–21. 10.1016/j.nicl.2016.05.006 27995067 PMC5153603

[27] Ebrahimian Dehaghani S Yadegari F Asgari A Bagheri Z. The mediator effect of cognition on the relationship between brain lesion location and dysphagia in patients with stroke: applying a structural equation model. J Oral Rehabil. (2019) 46:33–9. 10.1111/joor.12722 30252946

[28] Mesulam MM. A cortical network for directed attention and unilateral neglect. Ann Neurol. (1981) 10:309–25. 10.1002/ana.410100402 7032417

[29] Vaughn KA Archila-Suerte P Hernandez AE. Parietal lobe volume distinguishes attentional control in bilinguals and monolinguals: a structural MRI study. Brain Cogn. (2019) 134:103–9. 10.1016/j.bandc.2018.12.001 30528309

[30] Soros P Inamoto Y Martin RE. Functional brain imaging of swallowing: an activation likelihood estimation meta-analysis. Hum Brain Mapp. (2009) 30:2426–39. 10.1002/hbm.20680 19107749 PMC6871071

[31] Duhamel JR Colby CL Goldberg ME. Ventral intraparietal area of the macaque: congruent visual and somatic response properties. J Neurophysiol. (1998) 79:126–36. 10.1152/jn.1998.79.1.126 9425183

[32] Graziano MS Yap GS Gross CG. Coding of visual space by premotor neurons. Science. (1994) 266:1054–7. 10.1126/science.7973661 7973661

[33] Schroeder CE Lindsley RW Specht C Marcovici A Smiley JF Javitt DC. Somatosensory input to auditory association cortex in the macaque monkey. J Neurophysiol. (2001) 85:1322–7. 10.1152/jn.2001.85.3.1322 11248001

[34] Kern MK Jaradeh S Arndorfer RC Shaker R. Cerebral cortical representation of reflexive and volitional swallowing in humans. Am J Physiol Gastrointest Liver Physiol. (2001) 280:G354–60. 10.1152/ajpgi.2001.280.3.G354 11171617

[35] Perkin GD Murray-Lyon I. Neurology and the gastrointestinal system. J Neurol Neurosurg Psychiatry. (1998) 65:291–300. 10.1136/jnnp.65.3.291 9728939 PMC2170222

[36] Meadows JC. Dysphagia in unilateral cerebral lesions. J Neurol Neurosurg Psychiatry. (1973) 36:853–60. 10.1136/jnnp.36.5.853 4753882 PMC494474

